# Impulsivity and compulsivity in gambling disorder and bulimic spectrum eating disorders: Analysis of neuropsychological profiles and sex differences

**DOI:** 10.1192/j.eurpsy.2023.2458

**Published:** 2023-10-19

**Authors:** María Lozano-Madrid, Roser Granero, Ignacio Lucas, Isabel Sánchez, Jéssica Sánchez-González, Mónica Gómez-Peña, Laura Moragas, Nuria Mallorquí-Bagué, Javier Tapia, Susana Jiménez-Murcia, Fernando Fernández-Aranda

**Affiliations:** 1 Clinical Psychology Department, University Hospital of Bellvitge – ICS, Barcelona, Spain; 2 Ciber Fisiopatologia Obesidad y Nutrición (CIBERobn), Instituto Salud Carlos III, Barcelona, Spain; 3 Departament de Psicobiologia i Metodologia, Universitat Autònoma de Barcelona, Barcelona, Spain; 4 Psychoneurobiology of Eating and Addictive Behaviors Group, Neurosciences Programme, Bellvitge Biomedical Research Institute (IDIBELL), Barcelona, Spain; 5 Department of Clinical Sciences, School of Medicine and Health Sciences, University of Barcelona, Barcelona, Spain; 6 Department of Psychiatry, Hospital de Mataró, Consorci Sanitari del Maresme, Mataró, Spain

**Keywords:** bulimic spectrum eating disorders, compulsivity, gambling disorder, impulsivity, sex differences

## Abstract

**Background:**

Gambling disorder (GD) and bulimic spectrum eating disorders (BSDs) not only share numerous psychopathological, neurobiological, and comorbidity features but also are distinguished by the presence of inappropriate behaviours related to impulsivity and compulsivity. This study aimed to emphasise the differences and similarities in the main impulsivity and compulsivity features between GD and BSD patients, and to analyse the potential influence of sex in these domains.

**Methods:**

Using self-reported and neurocognitive measures, we assessed different impulsive–compulsive components in a sample of 218 female and male patients (59 with BSD and 159 with GD) and 150 healthy controls.

**Results:**

We observed that GD and BSDs exhibited elevated levels of impulsivity and compulsivity in all the dimensions compared to healthy controls. Moreover, these disorders showed differences in several personality traits, such as high novelty seeking in GD, and low persistence and high harm avoidance in BSDs. In addition, patients with BSDs also displayed a trend towards greater impulsive choice than GD patients. Regarding sex effects, GD women presented higher overall impulsivity and compulsivity than GD men. Nevertheless, no sex differences were found in BSDs.

**Conclusions:**

Clinical interventions should consider these deficits to enhance their effectiveness, including adjunctive treatment to target these difficulties. Our findings also provide support to the relevance of sex in GD, which should also be considered in clinical interventions.

## Introduction

Gambling disorder (GD) and eating disorders (EDs) are mental conditions characterised by persistent maladaptive patterns of gambling behaviour and abnormal eating, respectively [1]. Despite representing very different entities with specific clinical symptoms, these disorders share numerous psychopathological, neurobiological, and comorbidity features [2–6]. For instance, both disorders are distinguished by elevated behavioural disinhibition [7, 8], great levels of negative urgency [9, 10], as well as difficulties in executive function [11] and emotion regulation [5]. At a neurobiological level, there is a dysregulation of the dopaminergic and serotonin systems, which are respectively involved in the reward and impulse control systems [12–15]. GD and BSDs also display high levels of co-occurrence [16–18] and usually overlap with the same pathologies, especially with impulse control disorders and substance abuse [19–23]. Finally, GD and EDs are distinguished by the presence of inappropriate behaviours related to impulsivity and compulsivity [10, 22, 24, 25], and are considered to map into the impulsive–compulsive spectrum, a continuum encompassing different neuropsychiatric conditions characterised by impairments in impulse control mechanisms [26, 27].

Recent research suggests that impulsivity (i.e., rapid unplanned responses performed without regard to their negative consequences) can be conceptualised as a multidimensional construct composed of three major domains: ‘choice impulsivity’, ‘response impulsivity’, and ‘impulsive personality traits’ [28, 29]. *Choice impulsivity* is understood as impulsive decision-making without neither planning nor regard for future consequences [28], and is usually measured with the Iowa Gambling Task (IGT) [30]. Numerous studies have shown decision-making impairments in all EDs, but especially in bulimic spectrum disorders (BSDs) [31–33], meaning higher impulsive choice in these ED subtypes. Similarly, decision-making deficits have been observed in GD [34, 35], mainly when taking risk–reward choices, in which GD patients display high difficulty in choosing the advantageous options [36].

Secondly, *response impulsivity* would reflect the inability to inhibit a strong motor response, meaning a low inhibitory control [28]. The Stroop Colour and Word Test (SCWT) [37, 38] has shown poor inhibitory control and high response impulsivity in patients with EDs, mainly in BSD patients [39, 40]. Inhibitory control deficits are particularly outstanding when examining stimuli related to the disorder (e.g., food consumption) [41], suggesting they may constitute a maintenance factor in BSDs [42]. GD has also been linked with decreased inhibitory control, especially in certain cognitively demanding situations (e.g., gambling activities) [43, 44].

*Impulsive personality traits* are the last form of impulsivity, and refer to dispositional tendencies towards impulsive behaviour [28]. Novelty seeking, assessed using the Temperament and Character Inventory-Revised (TCI-R) [45], is the trait most closely related to impulsivity, since it refers to the search for new stimuli and rewards. Among EDs, the highest levels of novelty seeking are displayed by patients with BSDs [46–48], reflecting a more impulsive personality. Similarly, high novelty seeking has been identified as an outstanding feature in GD [49, 50]. Lastly, another impulsive trait highlighted in GD and particularly in BSDs is low persistence [51], indicating lack of perseverance in the presence of adversities [45].

Similarly to impulsivity, compulsivity (i.e., repetitive maladaptive behaviours performed to avoid negative consequences) can be described as a multifactorial construct made up of different dimensions [52, 53]. *Cognitive flexibility* is one of the most relevant components of compulsivity. It refers to the ability to adapt cognitive strategies in response to feedback [54], and is commonly measured by the Wisconsin Card Sorting Task (WCST) [55]. Numerous studies have displayed poor cognitive flexibility in patients with BSDs [31, 56, 57] and GD [11, 58], reflecting high levels of cognitive rigidity and compulsivity in both disorders. On the other hand, due to the lack of psychometric tools to measure compulsivity, some authors have suggested the use of harm avoidance (TCI-R) to assess *compulsive traits* [54, 59]. In this regard, several studies have shown that BSD and GD patients display higher levels of harm avoidance than healthy controls (HCs) [46, 47, 60].

Despite the similarities between GD and BSDs, only a few studies have explored impulsivity and compulsivity comparing both disorders. Studies focused on personality traits observed that GD patients show higher novelty seeking than BSD patients [16, 22, 50, 51, 61], meaning a higher tendency to search for new stimuli and rewards. On the other hand, BSD patients display lower persistence [51] and higher harm avoidance [50, 51], indicating higher sensitivity and vulnerability to adversities. Only one research compared the neuropsychological performance of GD and BN patients [11], revealing that GD patients exhibit poorer cognitive flexibility, which is a higher difficulty in adapting cognitive strategies in response to feedback. Finally, although sex differences are especially highlighted in these pathologies at a clinical and neuropsychological level [62–66], most of the cited studies did not consider the effect of sex on their results, possibly leading to a bias.

Given that numerous impulsive and compulsive features overlap across GD and BSDs, there is a need for comparative studies which examine the differences and similarities between both. Thus, this study aimed to compare impulsivity and compulsivity dimensions among HCs and patients with BSDs and GD. Firstly, we hypothesised that both clinical groups would display abnormalities in these domains relative to HCs. Based on previous studies, we also expected to find some differences between GD and BSDs, such as lower cognitive flexibility and higher novelty seeking in GD, but lower persistence and higher harm avoidance in BSDs. Secondly, we aimed to examine sex effects on the impulsivity and compulsivity of GD and BSD patients.

## Methodology

### Participants

The final sample consisted of 368 participants (52.2% females) aged between 18 and 72 years old, of which 59 were diagnosed with a BSD (62.7% females) and 159 with a GD (11.6% females) according to the DSM-5 diagnostic criteria [1]. The BSD subsample comprised of bulimia nervosa (47.5%) and binge ED patients (52.5%). The control group was made up of 150 individuals without a lifetime mental disorder.

Participants included in the clinical groups were referred to assessment and treatment at the Eating Disorder Unit and the Pathological Gambling Unit of the Department of Clinical Psychology of the Bellvitge University Hospital in Barcelona. They were diagnosed by psychologists/psychiatrists with over 10 years’ experience by means of a semi-structured interview. Healthy controls were recruited via word-of-mouth and advertisements located in the same geographic area. As they arrived at the Eating Disorder and Pathological Gambling Units, they were interviewed by psychologists/psychiatrists to verify their eligibility for the study. They could not have a lifetime history of an ED, current obesity or any behavioural addiction. Healthy controls were also matched for age and education level with participants of the clinical groups.

All participants were informed about the research procedures and gave their informed consent in writing. Exclusion criteria were the following: (1) history of chronic medical illness or neurological condition that might affect cognitive function; (2) head trauma with loss of consciousness for more than 2 min; (3) learning disability or intellectual disability; (4) use of psycho-active medications or drugs; (5) age under 18.

### Procedure and assessment

All individuals who arrived at the hospital and were diagnosed with an ED or a GD were screened for the inclusion criteria. Those included in the study underwent a clinical and neuropsychological assessment within the first week of their outpatient treatment. The instruments included were specifically selected to cover a wide range of impulsivity and compulsivity domains (see Supplementary Table S1). Additional sociodemographic information was also taken.

### Instruments

The Spanish adaptation of the Symptom Checklist-90 Revised (SCL-90-R) [67, 68] assesses psychopathological symptoms grouped as follows: somatisation, obsessive-compulsive, interpersonal sensitive, depression, anxiety, hostility, phobic anxiety, paranoia, and psychotic. Additionally, this scale contains a global severity index (GSI) that measures overall psychological distress. Cronbach’s alpha in this study was good to excellent (between *a* = 0.77 for phobic anxiety and *a* = 0.93 for depressive symptoms).

The Spanish validation of the TCI-R [45, 69] is a reliable questionnaire composed of 240 items. It includes four dimensions of temperament (novelty seeking, harm avoidance, reward dependence, and persistence) and three of character (self-directedness, cooperativeness, and self-transcendence). In this study sample, Cronbach’s alpha was good to excellent (between *a* = 0.73 for novelty seeking and *a* = 0.89 for self-directedness).

The SCWT [37, 70] is a neuropsychological tool that evaluates inhibitory control. It comprises three different lists: a word list containing names of colours, a colour list that comprises letter Xs printed in colour, and a colour-word list composed of names of colours in a colour ink that does not match the written name. Three scores are calculated using the number of items correctly read from each list in 45 s. An additional ‘interference score’ is obtained using all three lists. Better capacity of inhibitory control is related to higher scores in the colour-word list and in the interference score.

The Wisconsin Card Sorting Test (WCST) [55] is a computerised set-shifting task which allows the assessment of cognitive flexibility. Participants have to sort each of the 128 cards provided in one of the four available decks. Each card presents a figure with different properties (colour, shape, and number). The aim is to select the correct deck considering one of the properties. When participants select a deck, feedback (‘correct’ or ‘incorrect’) is displayed, hence they can deduce the selection criteria. The rule changes after completing a category (10 consecutive correct sorts) or if this is not discovered after six trials. The task finishes when all 128 cards are sorted or after six categories are completed. The main variable related to compulsivity is perseverative errors (i.e., failures to change sorting strategy after negative feedback).

The IGT [30] is a computerised task designed to assess decision-making processes and impulsive choices [71]. The task involves 100 trials in which participants have to select one of the four presented decks (A, B, C, or D); afterwards, a specified amount of play money is awarded or subtracted. Two of the decks result in money wins (C, D), while the others result in losses (A, B). Participants are instructed to gather as much money as possible. The test score is computed by subtracting the number of times that participants selected the disadvantageous decks from the number of advantageous deck choices. Lower scores translate to impulsive decision-making.

### Statistical analysis

Statistical analysis was carried out with Stata16 for Windows [72]. Firstly, the frequency distribution of the sociodemographic and clinical variables was based on chi-square tests (*χ* ^2^) for categorical variables and analysis of variance (ANOVA) for quantitative measures. The effect size of the mean differences was tested through Cohen’s *d* coefficient, considering low-poor effect for |*d*| > 0.20, moderate-medium for *|d*| > 0.5, and large-high for *|d*| > 0.8 [73]. The effect size of the proportion differences was estimated through Cohen’s *h* coefficient, obtained as the difference of the arcsine transformation for the proportions observed in each group; it was interpreted with the same threshold ranges as Cohen’s *d* measure [74]. The comparison of the impulsivity and compulsivity measures was based on 3 × 2 ANOVA procedures, controlling for the covariates chronological age and education. Two between-subjects factors were defined: group (with three levels: HCs, BSDs, and GD) and sex (with two levels: women and men). In addition, Finner correction was used to control the increase in the Type I error due to the multiple statistical comparisons [75], which is a family-wise procedure that has demonstrated higher power than classical Bonferroni correction.

### Ethics

The study procedures were carried out in accordance with the Declaration of Helsinki. The Clinical Research Ethics Committee of Bellvitge University Hospital approved the study.

## Results

### Characteristics of the sample

Table 1 contains the comparison of the sociodemographic and clinical profile of the three groups. Statistical differences emerged for all the measures among all three groups, with most effect sizes within the moderate to high range (|*d*| > 0.50 or |*h*| > 0.50).

**Table 1.** Descriptive of the sample

	HC *n = 150*		BSD *n = 59*		GD *n = 159*		BSD *vs.* HC		GD *vs.* HC		BSD *vs.* GD	
	*n*	%	*n*	%	*n*	%	*p*	|*h*|	*p*	|*h*|	*p*	|*h*|
Sex												
Women	123	82.0	37	62.7	32	20.1	.003^a^	0.44	.001^a^	1.33^b^	.001^a^	0.90^b^
Men	27	18.0	22	37.3	127	79.9						
Education												
Primary	8	5.3	4	6.8	75	47.2	.001^a^	0.06	.001^a^	1.05^b^	.001^a^	0.99^b^
Secondary	85	56.7	17	28.8	52	32.7		0.57^b^		0.51^b^		0.08
University	57	38.0	38	64.4	32	20.1		0.53^b^		0.40		0.93^b^

	Mean	SD	Mean	SD	Mean	SD	*p*	|*d*|	*p*	|*d*|	*p*	|*d*|
Age (years-old)	30.67	13.68	35.24	11.08	41.10	11.34	.016^a^	0.37	.001^a^	0.83^b^	.002^a^	0.52^b^
Onset of disorder	–	–	25.81	11.43	29.61	11.27	–	–	–	–	.029^a^	0.33
Duration of disorder	–	–	7.98	4.73	5.23	5.28	–	–	–	–	.001^a^	0.55^b^
SCL-90-R somatisation	0.62	0.47	1.73	0.90	1.01	0.83	.001^a^	1.53^b^	.001^a^	0.57^b^	.001^a^	0.82^b^
SCL-90-R obsessive-comp.	0.76	0.56	1.88	0.98	1.25	0.80	.001^a^	1.40^b^	.001^a^	0.70^b^	.001^a^	0.70^b^
SCL-90-R Interp. sensitive	0.63	0.54	1.99	1.07	1.11	0.81	.001^a^	1.60^b^	.001^a^	0.70^b^	.001^a^	0.92^b^
SCL-90-R depressive	0.64	0.51	2.19	1.06	1.66	0.86	.001^a^	1.87^b^	.001^a^	1.45^b^	.001^a^	0.55^b^
SCL-90-R anxiety	0.48	0.42	1.66	1.00	1.09	0.80	.001^a^	1.54^b^	.001^a^	0.96^b^	.001^a^	0.62^b^
SCL-90-R hostility	0.44	0.51	1.36	0.97	0.96	0.81	.001^a^	1.19^b^	.001^a^	0.77^b^	.001^a^	0.45
SCL-90-R phobic anxiety	0.12	0.27	1.08	0.86	0.48	0.58	.001^a^	1.50^b^	.001^a^	0.78^b^	.001^a^	0.82^b^
SCL-90-R paranoid	0.53	0.54	1.40	0.91	1.00	0.78	.001^a^	1.16^b^	.001^a^	0.71^b^	.001^a^	0.47
SCL-90-R psychotic	0.29	0.31	1.36	0.85	0.93	0.70	.001^a^	1.68^b^	.001^a^	1.20^b^	.001^a^	0.55^b^
SCL-90-R GSI score	0.55	0.40	1.73	0.83	1.14	0.68	.001^a^	1.81^b^	.001^a^	1.05^b^	.001^a^	0.78^b^
SCL-90-R PST score	21.25	6.69	57.32	19.13	48.71	20.92	.001^a^	2.52^b^	.001^a^	1.77^b^	.001^a^	0.43
SCL-90-R: PSDI score	1.44	0.31	2.34	0.64	1.92	0.57	.001^a^	1.79^b^	.001^a^	1.06^b^	.001^a^	0.69^b^

BSD, bulimic spectrum disorders; GD, gambling disorder; HC, healthy controls; SD, standard deviation.

a Significant comparison (.05 level).

b Effect size into the range mild–moderate (|*h*| or |*d*| higher than 0.50) to high-large (|*h*| or |*d*| higher than 0.80).

### Comparison of the impulsivity and compulsivity measures

Table 2 includes the results of the 3 × 2 ANOVA comparing the impulsivity and compulsivity measures among the three groups, controlling for age and education. Multivariate tests showed a significant effect of the factor group in most variables. When single effects were estimated, differences between HCs and the clinical groups were found for most measures, except for the following: IGT block 1, SCWT interference, TCI-R reward dependence, and WCST conceptual. Comparisons between BSDs and GD reported differences only for TCI-R novelty seeking (higher in GD) and harm avoidance (higher in BSDs).

**Table 2.** Comparison of the impulsivity and compulsivity measures: 3 × 2 ANOVA adjusted for age and education

	HC		BSD		GD		Women		Men		Multivariate tests^b^			Post-hoc comparisons^b^		
	*n = 150*		*n = 59*		*n = 159*		*n = 192*		*n = 176*		Factor	Factor				
	*Mean*	*SD*	*Mean*	*SD*	*Mean*	*SD*	*Mean*	*SD*	*Mean*	*SD*	Group	Sex	Inter.	BSD vs. HC	GD vs. HC	BSD vs. GD
IGT Block 1	−1.17	7.58	−2.08	6.02	−2.07	8.11	−2.05	6.26	−1.49	4.80	.508	.468	.826	.355	.319	.998
IGT Block 2	0.85	9.23	−1.72	7.32	−0.19	9.87	−0.67	7.74	−0.04	6.44	.095	.497	.001^a^	.030^a^	.345	.231
IGT Block 3	4.34	10.60	0.47	8.41	1.23	11.33	1.50	8.15	2.52	8.21	.006^a^	.342	.211	.005^a^	.014^a^	.606
IGT Block 4	3.51	11.78	0.34	9.35	2.74	12.59	1.82	9.16	2.57	8.83	.110	.526	.010^a^	.037^a^	.583	.144
IGT Block 5	4.78	12.43	−0.76	9.86	1.24	13.28	1.10	9.82	2.41	9.09	.001^a^	.297	.903	.001^a^	.017^a^	.248
IGT Total	12.38	33.67	−3.75	26.71	2.97	35.99	1.69	28.21	6.04	24.29	.001^a^	.203	.020^a^	.001^a^	.019	.151
SCWT words	105.16	21.13	102.86	16.76	98.89	22.58	103.57	16.21	101.04	16.89	.045^a^	.237	.010^a^	.397	.013^a^	.177
SCWT colours	72.84	16.43	68.26	13.03	68.29	17.56	71.44	13.03	68.15	12.43	.025^a^	.049^a^	.030^a^	.030^a^	.020^a^	.988
SCWT words-colours	47.19	13.32	43.67	10.56	43.76	14.23	44.76	11.39	44.98	10.71	.039^a^	.869	.674	.040^a^	.031^a^	.962
SCWT interference	4.45	10.29	2.98	8.17	3.62	11.00	2.68	8.23	4.69	8.23	.517	.054	.312	.266	.497	.656
TCI-R Novelty seeking	101.39	17.84	103.97	14.15	110.17	19.07	104.86	13.72	105.50	13.60	.001^a^	.725	.262	.260	.001^a^	.013^a^
TCI-R harm avoidance	91.43	22.84	118.25	18.12	105.64	24.42	108.60	20.77	101.62	18.00	.001^a^	.003^a^	.520	.001^a^	.001^a^	.001^a^
TCI-R reward depen.	101.62	19.06	97.92	15.12	100.57	20.37	102.57	14.52	97.51	14.06	.319	.009^a^	.052	.132	.643	.319
TCI-R persistence	111.85	23.95	103.73	19.00	107.35	25.60	104.99	18.88	110.29	17.42	.026^a^	.030^a^	.287	.009^a^	.115	.278
WCST trials	88.93	24.86	100.81	19.72	100.74	26.57	99.08	20.30	94.57	20.90	.001^a^	.075	.576	.001^a^	.001^a^	.984
WCST persev. errors	8.99	14.24	14.99	11.29	14.90	15.22	14.57	11.04	11.35	11.30	.001^a^	.027^a^	.948	.001^a^	.001^a^	.964
WCST non-per. errors	10.52	16.07	15.21	12.74	16.78	17.17	16.24	13.86	12.10	12.28	.003^a^	.011^a^	.266	.023^a^	.001^a^	.481
WCST conceptual	64.49	20.17	62.95	16.00	60.30	21.56	59.77	16.17	65.39	14.29	.218	.006^a^	.820	.554	.081	.344
WCST categ. complet	5.60	2.11	4.94	1.67	4.83	2.25	4.76	1.79	5.49	1.55	.004^a^	.001^a^	.305	.016^a^	.003^a^	.715
WCST trials first categ.	16.30	32.80	22.04	26.02	30.87	35.06	31.00	31.14	15.14	19.00	.001^a^	.001^a^	.065	.174	.001^a^	.053

BSD, bulimic spectrum disorders; GD, gambling disorder; HC, healthy controls; SD, standard deviation.

a Significant parameter.

b *p*-values.

Regarding the sex factor, differences between men and women were obtained for all the WCST measures, except for WCST trials, with men showing a better performance relative to women. Compared to women, men achieved lower scores in SCWT colours, TCI-R harm avoidance, and reward dependence, but higher levels in TCI-R persistence.

Multivariate tests also displayed a significant interaction sex-by-group for some variables: IGT block 2 and 4, IGT total, SCWT words, and SCWT colours. Single effects were estimated (see Supplementary Table S2).

**Figure 1.**
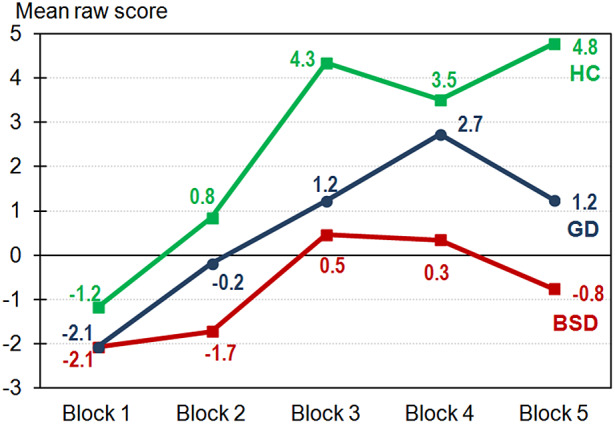
Learning curves in the IGT.

Figure 1 contains the learning curve of each group in the IGT, HCs reporting the best performance. When the two clinical groups were compared, GD displayed a trend towards better performance.

**Figure 2.**
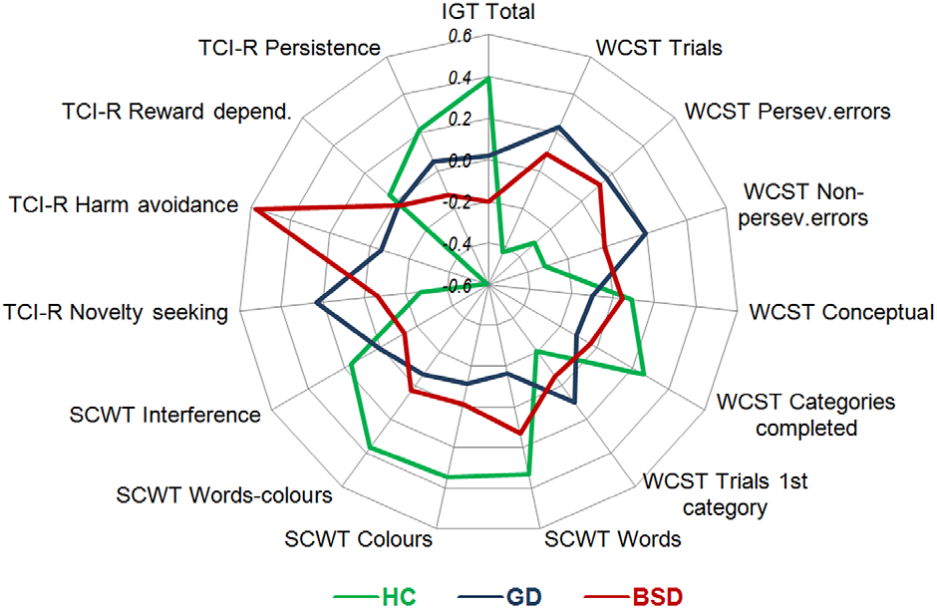
Summary of the results: radar chart (*z*-standardised mean scores). BSD, bulimic spectrum disorders; GD, gambling disorder; HC, healthy controls.

As a summary, Figure 2 illustrates the radar chart for the variables measuring impulsivity and compulsivity features (*z*-standardised means are plotted to allow an easier interpretation).

## Discussion

In this study we aimed to compare, through self-reported and neurocognitive measures, impulsivity and compulsivity among HCs, GD, and BSDs, emphasising the differences and similarities between the two clinical groups. We also aimed to analyse the potential influence of sex in these impulsive and compulsive domains.

Firstly, when exploring general psychopathology, we observed notable differences among the three groups in all the SCL-90-R subscales. In agreement with previous studies [16, 22, 51], HCs exhibited the lowest levels of global psychopathology and specific symptomatology, while BSD patients displayed the highest levels. This could be interpreted as an increased perception of psychopathological distress in BSDs.

Regarding *choice impulsivity*, our results illustrated that GD and BSD patients had worse IGT performance than HCs, indicating a reduced capacity to learn the reward/punishment contingencies of their choices [76]. In addition, BSD patients performed slightly worse than patients with GD, demonstrating a trend to more impulsive decision-making. Attending to earlier findings, it was expected that both clinical groups presented difficulties in decision-making [31, 32, 48, 77]. Evidence also indicates that GD and BSD patients share a preference for immediate rewards (i.e., binging or gambling) regardless of future consequences [78, 79]. This might reflect their increased choice impulsivity, which appears to be slightly enhanced in BSDs. When comparing women and men, significantly increased choice impulsivity was displayed by women only in GD, although BSD women also showed a trend to higher choice impulsivity than men. Previous studies failed to find sex differences in the decision-making of AN patients [57], indicating that sex influences might be only noticeable in ED patients with binge-purging symptoms. Regarding GD, several metanalyses failed to find any sex effect on this domain [77, 80], which is likely to be explained by methodological issues (e.g., metanalyses compared female samples to male samples). We also observed that men exhibited similar choice impulsivity regardless of the diagnostic group; however, among women, both clinical groups displayed higher choice impulsivity than HCs. Altogether, our results indicate that enhanced choice impulsivity might be highlighted only in female patients.

Another dimension of impulsivity examined in this work was *response impulsivity.* Our results revealed that both clinical groups showed lower inhibitory control than HCs, as observed in the SCWT word-colour scores. Recent research also noted significant inhibitory control deficits in GD and BSDs [39, 40, 44], and the only study which compared both disorders also found analogous outcomes [11]. These similarities in response impulsivity found in GD and BSDs may be related to common underlying processes involving impairments in behavioural inhibition systems [3]. Finally, women and men did not differ in inhibitory control, neither in the whole sample nor within each clinical group. Although a recent study has shown lower inhibitory control in women with BSDs [66], there is very little evidence to examine these effects in GD, presenting similar evidence to this work [64, 80].

The results of this work also illustrated that, in terms of *impulsive personality traits*, patients with GD showed the highest levels of novelty seeking in the TCI-R, differing from BSD patients and HCs. In addition, BSD patients showed lower levels of persistence compared to HCs. It is noted that, although BSDs are considered to be the EDs with the highest impulsive personality [46, 47], BSD patients displayed similar novelty seeking to HCs. Our finding is consistent with former investigations [47, 51, 60, 61] and suggests that novelty seeking might not be a core factor of impulsive personality in BSDs; other traits, such as low persistence, seem to be more relevant. As a whole, these findings give support to previous research [16, 22, 50, 51, 61] and uphold the notion that impulsive personality in GD and BSDs appears to be from a different nature, with high novelty seeking as the distinctive trait in GD, and low persistence in BSDs. Concerning sex, no differences in novelty seeking were found within the clinical groups, although lower persistence was displayed by women in the GD group. Prior studies in GD and EDs partially support our findings, since no sex differences were yielded in any of these impulsive traits [62, 63]. In addition, although earlier studies reported novelty-seeking differences between GD and BSDs in both sexes [16, 22, 60, 61], our study only displayed differences among men. This might indicate that, regarding impulsive traits, GD and BSDs may be more closely associated in females than in males. However, future research exploring sex differences in these disorders would be required to make solid assumptions.

Another finding to emerge from the present study are the differences in *cognitive flexibility.* Compared to HCs, patients with GD and BSDs displayed a worse performance in most measures of the WCST, including perseverative errors, meaning less cognitive flexibility and more compulsivity. This outcome is in line with previous research [11, 31, 56, 58, 81–83] and might explain the inability of GD and BSD patients to learn from mistakes and handle negative affect, increasing loss of control over gambling or eating [84–87]. Contrary to our hypothesis, we did not succeed in identifying greater cognitive flexibility impairments in GD compared to BSDs. A former work had revealed such results [11], although they were considered preliminary, due to the fact that the sample was considerably small and only included females. Since comparative studies of compulsivity in BSDs and GD are extremely scarce, solid conclusions still cannot be made. Focusing on sex effects, worse cognitive flexibility in females than males was observed in the whole sample. Nevertheless, when examining each diagnostic group, this pattern only remained present in the GD group. Only a few studies had explored sex differences in compulsivity between women and men with BSDs and GD, showing women lower cognitive flexibility in both pathologies [64, 66]. Moreover, when comparing GD and BSDs within each sex, the clinical groups showed lower cognitive flexibility than HCs in both sexes. As a whole, these results reveal that females with GD appear to have more compulsivity features in common with BSD females than with GD males.

Regarding *compulsive personality traits*, differences in harm avoidance were observed among all three groups, with BSD patients showing the highest scores, followed by GD patients. Our findings dovetail with previous reports and uphold that compulsive traits are a distinguishing feature in these pathologies [60], especially in BSDs [48, 50, 51]. Therefore, the elevated compulsivity noted in these pathologies is likely to underlie the use of abnormal behaviours (e.g., binge eating, compensatory purging, gambling) with the aim of avoiding negative affect and relieving emotional distress [24, 25]. Finally, when we explored sex effects on harm avoidance, higher scores were displayed by women in the overall sample and within the GD group. These results are in line with previous research [63] and with our findings in cognitive flexibility, indicating that GD women may differ from GD males and present a specific profile characterised by higher compulsivity. These findings could be indirectly related to the increased emotion regulation difficulties found in women with GD when compared to male patients [88]. Finally, differences among all three groups emerged when exploring women and men separately. As observed in the overall sample, the highest harm avoidance was displayed by BSDs, followed by GD. These results indicate that high levels of compulsive traits are noticeable in both GD and BSD patients regardless of their sex, being significantly highlighted in female and male patients with BSDs.

These findings should be interpreted considering some limitations. Firstly, our study sample comprised mostly young adults, not allowing for generalisation to older populations. In addition, since the age of participants could be a relevant factor affecting impulsivity and compulsivity in these disorders, it would be important to collect bigger samples and explore this point in future studies. Secondly, although results have been controlled for age and education, other confounding variables might be affecting. For instance, it would be useful to measure severity level following the DSM 5 criteria: mild, moderate, and severe. Having a bigger sample would allow us to find out if severity could be interacting with the variables studied (type of disorder and gender) and affecting impulsivity/compulsivity levels. Moreover, the subgroup sizes are relatively large but not evenly distributed due to the sex bias usually observed in these disorders. A bigger sample would allow us to reduce this bias and create subgroups more equally distributed. Lastly, the cross-sectional nature of this study does not allow for cause–effect inferences. Longitudinal studies are needed to explore if impulsivity and compulsivity deficits are a cause or a consequence of the disorder.

## Conclusions

In conclusion, GD and BSDs seem to be distinguished by elevated levels of impulsivity and compulsivity in all the dimensions, showing differences in some domains mainly related to personality. In this regard, these pathologies show distinct impulsive personality traits, such as high novelty seeking in GD and low persistence in BSDs. Moreover, BSDs are characterised by higher compulsive personality traits as well as a trend towards greater impulsive choice. Given the lack of neuropsychological and clinical studies comparing GD and BSDs, future research would be needed to consolidate our findings. Nevertheless, clinical interventions should consider these deficits to enhance their effectiveness, for instance, including adjunctive treatment to target these difficulties, such as inhibitory control training [89], emotion regulation training [90, 91], or cognitive remediation therapy [92]. Finally, it seems that GD women present higher difficulties than GD men in almost all the impulsivity and compulsivity components, displaying in some cases more similarities with BSD women than with GD men. This finding is in line with former research providing support to the relevance of sex in GD [93], which should also be considered in clinical interventions. For instance, GD women would benefit from a more personalised approach which targets impulse control deficits, combining pharmacological treatment, cognitive behavioural therapy, family involvement, and support groups [94].

## Supporting information

Lozano-Madrid et al. supplementary material Lozano-Madrid et al. supplementary material

Lozano-Madrid et al. supplementary material Lozano-Madrid et al. supplementary material

## Supplementary material

The supplementary material for this article can be found at https://doi.org/10.1192/j.eurpsy.2023.2458.
