# Supplementary material for: Impulsivity and compulsivity in gambling disorder and bulimic spectrum eating disorders: Analysis of neuropsychological profiles and sex differences
Source: Eur Psychiatry. 2023 Oct 19;66(1):e91. doi: 10.1192/j.eurpsy.2023.2458 (PMC10755579; doi:10.1192/j.eurpsy.2023.2458)
Supplement: Lozano-Madrid et al. supplementary material [file S0924933823024586sup001.docx]

**SUPPLEMENTARY MATERIAL**

***Table S1.*** Assessment of the different components of impulsivity and compulsivity

|  | Specific component | Cognitive/Personality domain | Measures |
| --- | --- | --- | --- |
| Impulsivity | Choice impulsivity | Decision-making | IGT |
|  | Response impulsivity | Inhibitory control | SCWT |
|  | Impulsive traits | Novelty seeking | TCI-R |
| Compulsivity | Cognitive flexibility | Attentional set-shifting | WCST |
|  | Compulsive traits | Harm avoidance | TCI-R |

Note. IGT: Iowa Gambling Task. SCWT: Stroop Colour and Word Test. TCI-R: Temperament and Character Inventory Revised. WCST: Wisconsin Card Sorting Test.

***Table S2.*** Post-hoc comparisons (*p*-value) for the interaction sex-by-group adjusted for age and education

|  | HC | | | | BSD | | | | GD | | | | Group within sex | | | | | | Sex within group | | |
| --- | --- | --- | --- | --- | --- | --- | --- | --- | --- | --- | --- | --- | --- | --- | --- | --- | --- | --- | --- | --- | --- |
|  | Women | | Men | | Women | | Men | | Women | | Men | | Within women | | | Within men | | | Within | Within | Within |
|  | *n=123* | | *n=27* | | *n=37* | | *n=22* | | *n=32* | | *n=127* | | BSD  vs. HC | GD  vs. HC | BSD  vs. GD | BSD  vs. HC | GD  vs. HC | BSD  vs. GD | HC  W-M | BSD  W-M | GD  W-M |
|  | *Mean* | *SD* | *Mean* | *SD* | *Mean* | *SD* | *Mean* | *SD* | *Mean* | *SD* | *Mean* | *SD* |  |  |  |  |  |  |  |  |  |
| IGT Block 1 | -1.12 | 6.71 | -1.23 | 6.13 | -2.44 | 5.86 | -1.71 | 5.67 | -2.61 | 6.17 | -1.54 | 6.29 | .222 | .273 | .912 | .775 | .814 | .900 | .932 | .633 | .359 |
| IGT Block 2 | 3.54 | 8.17 | -1.84 | 7.47 | -3.62 | 7.13 | 0.17 | 6.91 | -1.94 | 7.51 | 1.57 | 7.66 | **.001*** | **.001*** | .360 | .323 | **.031*** | .396 | **.002*** | **.042*** | **.013*** |
| IGT Block 3 | 5.14 | 9.38 | 3.53 | 8.58 | -0.28 | 8.19 | 1.21 | 7.94 | -0.36 | 8.62 | 2.82 | 8.79 | **.001*** | **.004*** | .967 | .320 | .693 | .396 | .416 | .486 | **.050*** |
| IGT Block 4 | 5.87 | 10.42 | 1.14 | 9.53 | -1.51 | 9.10 | 2.19 | 8.82 | 1.08 | 9.58 | 4.39 | 9.77 | **.001*** | **.024*** | .268 | .684 | .107 | .296 | **.031*** | .119 | .067 |
| IGT Block 5 | 4.04 | 10.99 | 5.53 | 10.05 | -1.72 | 9.60 | 0.20 | 9.30 | 0.97 | 10.10 | 1.51 | 10.30 | **.001*** | .169 | .276 | .052 | .059 | .556 | .519 | .443 | .778 |
| IGT Total | 17.4 | 29.8 | 7.3 | 27.2 | -9.6 | 26.0 | 2.1 | 25.2 | -2.8 | 27.4 | 8.8 | 27.9 | **.001*** | **.001*** | .312 | .479 | .801 | .266 | .106 | .087 | **.025*** |
| SCWT Words | 108.9 | 18.7 | 101.5 | 17.1 | 106.4 | 16.3 | 99.3 | 15.8 | 95.5 | 17.2 | 102.3 | 17.5 | .415 | **.001*** | **.009*** | .647 | .811 | .428 | .060 | .097 | **.034*** |
| SCWT Colours | 75.1 | 14.5 | 70.5 | 13.3 | 72.3 | 12.7 | 64.2 | 12.3 | 66.9 | 13.4 | 69.7 | 13.6 | .221 | **.005*** | .100 | .082 | .758 | .064 | .133 | **.016*** | .271 |
| SCWT Words-colours | 47.2 | 11.8 | 47.2 | 10.8 | 44.3 | 10.3 | 43.1 | 10.0 | 42.9 | 10.8 | 44.7 | 11.0 | .125 | .070 | .596 | .162 | .266 | .507 | .995 | .665 | .374 |
| SCWT Interference | 2.86 | 9.11 | 6.05 | 8.33 | 1.42 | 7.95 | 4.55 | 7.70 | 3.76 | 8.37 | 3.49 | 8.53 | .329 | .626 | .252 | .508 | .146 | .563 | .096 | .132 | .863 |
| TCI-R Novelty seeking | 98.8 | 15.8 | 103.9 | 14.4 | 105.0 | 13.8 | 103.0 | 13.3 | 110.8 | 14.5 | 109.6 | 14.8 | **.017*** | **.001*** | .100 | .807 | .066 | **.039*** | .124 | .582 | .654 |
| TCI-R Harm avoidance | 93.0 | 20.2 | 89.9 | 18.5 | 122.3 | 17.6 | 114.2 | 17.1 | 110.5 | 18.6 | 100.8 | 18.9 | **.001*** | **.001*** | **.001*** | **.001*** | **.005*** | **.001*** | .460 | .079 | **.006*** |
| TCI-R Reward dependence | 101.1 | 16.9 | 102.1 | 15.4 | 103.7 | 14.7 | 92.2 | 14.3 | 102.9 | 15.5 | 98.2 | 15.8 | .353 | .596 | .849 | **.018*** | .230 | .078 | .778 | **.003*** | .106 |
| TCI-R Persistence | 111.7 | 21.2 | 112.0 | 19.4 | 100.9 | 18.5 | 106.5 | 17.9 | 102.3 | 19.5 | 112.4 | 19.9 | **.002*** | **.030*** | .767 | .302 | .928 | .174 | .950 | .246 | **.007*** |
| WCST Trials | 90.5 | 22.0 | 87.4 | 20.1 | 101.9 | 19.2 | 99.7 | 18.6 | 104.9 | 20.2 | 96.6 | 20.6 | **.001*** | **.001*** | .545 | **.025*** | **.030*** | .482 | .505 | .666 | **.030*** |
| WCST Perseverative errors | 10.6 | 12.6 | 7.4 | 11.5 | 16.3 | 11.0 | 13.7 | 10.7 | 16.8 | 11.6 | 13.0 | 11.8 | **.005*** | **.015*** | .859 | **.045*** | **.022*** | .788 | .228 | .359 | .081 |
| WCST Non-persev. errors | 12.1 | 14.2 | 8.9 | 13.0 | 15.9 | 12.4 | 14.5 | 12.0 | 20.7 | 13.1 | 12.9 | 13.3 | .096 | **.003*** | .135 | .117 | .153 | .573 | .291 | .655 | **.002*** |
| WCST Conceptual | 61.8 | 17.8 | 67.1 | 16.3 | 60.8 | 15.6 | 65.1 | 15.1 | 56.6 | 16.4 | 64.0 | 16.7 | .729 | .148 | .291 | .643 | .363 | .765 | .160 | .300 | **.017*** |
| WCST Categories completed | 5.31 | 1.86 | 5.88 | 1.71 | 4.74 | 1.63 | 5.15 | 1.58 | 4.24 | 1.71 | 5.43 | 1.75 | .057 | **.005*** | .237 | .114 | .208 | .455 | .144 | .331 | **.001*** |
| WCST Trials 1^st^ category | 20.9 | 29.0 | 11.7 | 26.5 | 27.7 | 25.3 | 16.3 | 24.5 | 44.4 | 26.7 | 17.4 | 27.2 | .147 | **.001*** | **.011*** | .519 | .311 | .860 | .131 | .086 | **.001*** |

*Note.* SD: standard deviation. HC: healthy controls. BSD: bulimic spectrum disorders. GD: gambling disorder. *Bold: significant parameter.
